# Continuing professional development training needs for primary care doctors in central Uganda

**DOI:** 10.4102/phcfm.v15i1.3983

**Published:** 2023-10-18

**Authors:** Jane Frances Namatovu, William Buwembo, Janet Nakigudde, Sarah Kiguli, Aloysius G. Mubuuke

**Affiliations:** 1 Department of Family Medicine, Faculty of Medicine, Makerere University, Kampala, Uganda; 2 Department of Anatomy, Faculty of Biomedical Sciences, Makerere University, Kampala, Uganda; 3 Department of Psychiatry, Faculty of Medicine, Makerere University, Kampala, Uganda; 4 Department of Pediatrics and Child Health, Faculty of Medicine, Makerere University, Kampala, Uganda; 5 Health Profession’s Education Unit, Faculty of Medicine, Makerere University, Kampala, Uganda; 6 Department of Radiology, Faculty of Medicine, Makerere University, Kampala, Uganda

**Keywords:** training needs analysis, primary care doctors, continuing professional development, district health system, central Uganda, developing countries

## Abstract

**Background:**

Continuing professional development (CPD) activities relevant to medical doctors and their patients should be informed by current assessed training needs. The CPD provision is expected to improve the quality of professional practice and ethics. However, the Uganda Medical and Dental Practitioners’ Council still receives about 40 reports of malpractice every month.

**Aim:**

The study aimed to describe the CPD training needs of doctors working in public primary care facilities in central Uganda.

**Setting:**

The district health system of central Uganda comprised 10 General Hospitals (GH) and 37 Health Center IVs (HC IVs) with a staffing norm of six and two doctors, respectively.

**Methods:**

This was a cross-sectional survey of 100 doctors working in public primary care facilities using the World Health Organization (WHO) Hennessy-Hicks questionnaire. Descriptive statistics of the importance, current performance, and training need of each skilled activity were calculated. Content analysis was applied to data from the open-ended questions.

**Results:**

The response rate was 91%, majority were males, 80 (87.9%) from 7 GHs and 24 HC IVs with an average age of 37.9 years. The domain with the highest CPD training need for the doctors was research and audit, with a mean score (standard deviation [s.d.]) of 1.94 (±1.69), followed by administration 1.58 (±1.61) and clinical tasks 1.28 (±1.29). The clinical tasks domain had the most suggested CPD topics.

**Conclusion:**

Research and audit and clinical tasks were identified as important domains for CPD training for doctors in this setting.

**Contribution:**

The results give insight into CPD training needs of primary care doctors and guide various CPD providers.

## Introduction

Continuing professional development (CPD) is the process by which health professionals keep updated to meet the current needs of patients, the health services, and their professional development.^1,2^ A well-functioning and responsive primary care sector demands for doctors to be well supported, through CPD in clinical, educational and leadership roles.^3^ Effective CPD has been found to improve the quality of primary care services provided.^4^ Continuing professional development activities that are relevant to the doctors and their patients should be informed by the current assessed training needs specific to the context.^1,5,6,7^ The training needs depend on the context in which the doctors practice,^5,8^ that is where they encounter several learning opportunities and challenges.^9^

Primary care in Uganda is provided within the district health system, which comprises Health Center I (Village Health Teams) to Health Center IV (HC IV) and the General Hospitals (GH). In the public primary care facilities, medical doctors are deployed at HC IV and GH levels. They provide frontline first-contact promotive, preventive, curative, rehabilitative, and palliative care.^10^ They also provide leadership for the primary care teams in the district health system.^11,12^ Therefore, a broad set of competencies need to be maintained by relevant CPD activities informed by identified training needs.

Despite Uganda’s high disease burden, the healthcare workforce at primary care level is inadequate in numbers.^13^ Relevant and focussed CPD activities may contribute to effectively utilising healthcare’s limited economic and personnel resources to improve primary care services. Uganda has also been reported as one of the countries with inadequate health worker performance with poor quality of primary care services.^14^ These services cater for the majority of the population in need, but their quality is affected by preventable medical errors that are partially because of poor performance of personnel because of insufficient technical skills.^15,16^

All Ugandan doctors are governed by the Uganda Medical and Dental Practitioners’ Council (UMDPC) whose mission is to regulate and enforce standards of education and practice to protect society from the harmful effects of malpractice. In 2017, UMDPC accredited several CPD providers^17^ and set up guidelines^18^ to improve effectiveness. This has not yet been realised because the UMDPC receives about 40 reports of malpractice every month.^19^ The reports are received by the registrar of the UMDPC in the form of letters of complaints signed by the complainant or their legal representative to follow the set guidelines.^20^ The commonest form of malpractice reported is practitioners’ violation of patients’ rights and negligence of their duties. The practitioners who are found guilty by the council are warned, put on probation or removed from the council’s register.^19^ The current CPD activities are mainly CPD-provider centred, with limited or no input from the primary care doctors. Studies conducted in other regions of the world have found that CPD training needs of primary care doctors included: understanding the health system, ability to be change agents, personal well-being and caring for colleagues, antibiotic treatment, surgery and emergency care.^21,22^ The purpose of this study, therefore, was to assess and describe the CPD training needs for primary care doctors that will inform CPD efforts in the region.

## Research method and designs

### Study design

A cross-sectional study was conducted to determine the CPD training needs of doctors working in public GHs and HC IVs in central Uganda.

### Setting

Uganda is divided into four health regions: central, eastern, northern, and western. Central Uganda had a projected population of 11.5 million by 2020. The central region is subdivided into three sub-regions by the Ministry of Health: north-central, south-central, and Kampala.^23^ In the public sector, this region has 10 GHs and 37 HC IVs. According to the 2016 Uganda Ministry of Health, guidelines for the local government planning process health sector supplement, the GH and HC IV have staffing norms of up to six and two doctors, respectively. But the UMDPC register reflects only up to 100 doctors in the public primary healthcare facilities of this region.

### Study population and sampling strategy

Primary care providers include the following health worker categories: medical doctors, dental surgeons, nurses and midwives, pharmacists, and allied health professionals.^24^ The study population comprised 100 doctors with an MBChB degree or its equivalent on the UMDPC register employed in public GHs and HC IVs found in the 26 districts of central Uganda. The study included medical doctors who had worked in the central region for at least 6 months or more in a public GH or HC IV. A consecutive sampling strategy was used to recruit the doctors to participate in the study. The email and phone contact details of the doctors were accessed from the UMDPC register with permission. The doctors were then contacted on phone to request for their participation in the study and preferred mode of delivery of the consent form and self-administered study questionnaire.

### Data collection

Data were collected using the validated Hennessy-Hicks training needs assessment (TNA) questionnaire.^7,25^ The questionnaire, which is in English, has been used in Uganda before to assess the training needs of other health workers.^26^ The consent form and questionnaire were emailed to all the 100 doctors or delivered physically at the health facility. The mode of delivery depended on the doctor’s preference after accepting to participate in the study. The participants were given up to 2 weeks to return the completed questionnaire, with weekly reminders by phone call. On completion, participants who accepted to use the email sent back the signed consent form and completed questionnaire, whereas those who received them physically gave a collection date that was effected. The respondents signed written consent were separated from the questionnaire to ensure anonymity. Data collection was carried out between January 2021 and October 2021.

### The Hennessy-Hicks training needs assessment questionnaire

The Hennessy-Hicks TNA questionnaire,^25^ is a widely used validated tool for assessment and evaluation of training needs for professional continuing education programmes across the healthcare workforce.^7^ It was applicable for this study because of the clinical practice setting (primary care) and educational nature of the study.^7^ The self-administered questionnaire consists of closed and open questions obtaining quantitative and qualitative data investigating CPD training needs of the primary care doctors. The self-administered questionnaire consists of two sections. Section 1 consists of questions that belong to one of the five main subsections. The subsections include: seven items for research and audit, six items for communication and teamwork, eight items for clinical tasks, three items for administration, and nine items for management and supervisory tasks. Each item is rated using a seven-point scale on its importance to the respondent (rate A) and the current performance of the skill by the respondent (rate B). In section 2 of the questionnaire, the respondents were asked to list up to 10 specific CPD training needs relevant to their current medical practice and work environment.

### Data analysis

All data were handled with strict confidentiality that included capturing, cleaning, and analysis. The data were entered into and cleaned using EPIDATA version 3.2. It was then exported to Statistical Package for Social Sciences (SPSS) version 12.0 (Chicago, United States of America) for analysis. Two categories of CPD training needs were assessed: CPD training needs based on the TNA using a quantitative descriptive survey (section 1) and specific CPD training needs for doctors in primary care using an open-ended question (section 2). Descriptive statistics were computed and summarised for the demographics. The mean scores at 95% confidence intervals (CIs) for the importance (A) and current performance (B) of each skilled activity were computed. The training need for each item was then calculated by subtracting mean scores of current performance (B) from mean scores of importance (A). An overall score per domain was thereafter calculated to rank its importance. Section 2 of the questionnaire was analysed using content analysis and frequencies of the desired CPD training needs were recorded. Responses were coded and grouped by the principal investigator into broad categories of tasks that need CPD training with reference to the work environment. The co-investigators reviewed the coding and confirmed that the domains were a true representation of the analysed data. The frequency of the overall score per activity in each domain was then calculated and recorded.

### Ethical considerations

Ethical approval to conduct the study was granted by the School of Medicine Research and Ethics Committee (REC REF 2019-126) at Makerere University College of Health Sciences and the Uganda National Council for Science and Technology (HS1170ES). Permission was obtained from the UMDPC to use the current national doctors register to get the doctors’ contact details. The doctors were contacted by telephone before a consent form was sent or delivered to them requesting for their participation. Participants were also informed of their right to withdraw from the study at any time without any negative consequences.

## Results

The study recruited 91 of the 100 doctors eligible on the UMDPC register (91% response rate) from 7 GHs and 24 HC IVs, with only 39 (42.8%) using the online option to respond to the questionnaire. Forty-four (48.4%) doctors reported working in GHs, while 47 (51.6%) worked in HC IVs. The average age of the respondents was 37.9 years (±7.9 years), ranging from 26 to 58 years. The majority of the respondents were males, 80 (87.9%), and the average number of years in the workplace was 4 years (±5.13 years). The respondents had been in their current posts for varied times: 6 months to 2 years (27, 29.7%); 3–5 years (28, 30.8%); 6–10 years (23, 25.2%); and more than 10 years (13, 14.3%). Most respondents (54, 59.3%) had some form of additional training after their basic degree in medicine.

Table 1 summarises the ratings for the different skilled activities with respect to importance and current performance for the 33 items used in this study. Considering the importance of the tasks in the primary care settings, it was revealed that communication and teamwork was the most important domain, with a mean score (standard deviation [s.d.]) of 6.63 (±0.61) followed by management or supervisory and clinical tasks. Similarly, the current performance of the tasks was best in the communication and teamwork domain with a mean score (s.d.) of 5.83 (±0.79).

**TABLE 1:** Skilled activities by importance, current performance and continuing professional development training need.

Sub-sections	Skilled activity	Importance			Current performance			CPD training need		
		Mean	s.d.	95% CI	Mean	s.d.	95% CI	Mean	s.d.	95% CI
Communication and teamwork	Communication with patients	6.81	0.77	6.65–6.97	6.38	0.90	6.20–6.57	0.43	1.22	0.17–0.68
	Getting on with colleagues	6.84	0.520	6.73–6.94	6.40	0.91	6.21–6.58	0.44	1.08	0.22–0.66
	Providing feedback to colleagues	6.56	0.96	6.36–6.76	5.54	1.29	5.27–5.81	1.02	1.36	0.74–1.30
	Negotiation and conflict resolution skills	6.40	1.04	6.18–6.61	5.12	1.61	4.79–5.46	1.28	1.71	0.92–1.63
	Giving clear advice or instructions to patients	6.82	0.46	6.73–6.92	6.30	1.07	6.07–6.52	0.53	1.21	0.28–0.79
	Patient advocacy and social duty as a doctor	6.34	1.24	6.08–6.60	5.22	1.74	4.86–5.58	1.12	1.72	0.76–1.48
	Total	6.63	0.61	6.50–6.75	5.83	0.79	5.66–5.99	0.80	0.97	0.59–1.00
Administration	Good record keeping or data input	6.71	0.92	6.52–6.91	5.30	1.48	4.99–5.60	1.42	1.92	1.02–1.82
	Use of new media and digital technology	6.32	1.14	6.08–6.56	4.33	1.85	3.94–4.72	1.99	2.25	1.53–2.46
	Communication with the District Health Office (DHO) or regulatory bodies, for example, UMDPC and professional bodies, for example, Uganda Medical Association (UMA)	6.41	1.27	6.14–6.67	5.08	1.78	4.71–5.45	1.33	1.89	0.93–1.72
	Total	6.48	0.89	6.29–6.66	4.90	1.20	4.65–5.15	1.58	1.61	1.24–1.91
Management or Supervisory tasks	Appraising your own performance or conducting audit	6.58	0.82	6.41–6.75	5.24	1.69	4.89–5.59	1.34	1.94	0.94–1.75
	Introducing new ideas or services or clinics at work	6.32	1.13	6.08–6.55	5.15	1.56	4.83–5.48	1.16	1.75	0.80–1.53
	Teaching or training colleagues, staff or students how to do things	6.73	0.62	6.60–6.85	5.85	1.32	5.57–6.12	0.88	1.41	0.59–1.17
	Organising and managing your time and workload	6.56	0.91	6.37–6.75	5.48	1.40	5.19–5.78	1.08	1.51	0.76–1.39
	Resource management or business skills or generating income or practice management	6.12	1.49	5.81–6.43	4.20	1.99	3.78–4.61	1.92	2.53	1.39–2.45
	Working as a member of the team	6.69	0.88	6.51–6.88	6.07	1.36	5.78–6.35	0.62	1.29	0.36–0.89
	Coping with and managing change in the healthcare service	6.51	0.91	6.32–6.70	5.49	1.50	5.18–5.81	1.01	1.68	0.66–1.36
	Stress management (self)	6.66	0.93	6.46–6.85	5.10	1.84	4.72–5.48	1.56	2.15	1.11–2.01
	Leadership skills	6.66	0.86	6.48–6.84	5.65	1.47	5.34–5.95	1.01	1.74	0.65–1.37
	Total	6.54	0.69	6.39–6.68	5.36	0.90	5.17–5.55	1.18	1.23	0.92–1.43
Clinical tasks	Procedural skills	6.77	0.75	6.61–6.92	5.93	1.32	5.66–6.21	0.84	1.59	0.50–1.17
	Recognising and managing risk in clinical practice	6.68	0.79	6.52–6.85	5.78	1.25	5.52–6.04	0.90	1.29	0.63–1.17
	Doing procedures or using new equipment or technology	6.07	1.44	5.77–6.37	4.18	1.92	3.78–4.58	1.89	2.53	1.36–2.42
	Searching medical literature or online healthcare resources	6.58	0.75	6.43–6.74	5.29	1.69	4.93–5.64	1.29	1.77	0.93–1.66
	Planning and organising individual patient care	6.69	0.71	6.54– 6.84	5.85	1.18	5.60–6.09	0.85	1.27	0.58–1.11
	Evaluating patients’ psychological and social needs	6.52	0.89	6.33–6.70	4.52	1.88	4.12–4.91	2.00	2.16	1.55–2.45
	Practicing health promotion and preventive medicine	6.62	0.93	6.42–6.81	5.38	1.66	5.04–5.73	1.23	1.67	0.88–1.58
	Prescribing healthy lifestyle and weight management	6.36	1.39	6.07–6.65	5.15	1.87	4.76–5.54	1.21	1.88	0.82–1.60
	Total	6.54	0.70	6.39–6.68	5.26	1.05	5.04–5.48	1.28	1.29	1.01–1.55
Research and audit	Safe and evidence-based prescribing	6.87	0.48	6.77–6.97	6.05	1.08	5.83–6.28	0.81	1.18	0.57–1.06
	Interpreting your own research findings	5.98	1.73	5.62–6.34	4.58	2.09	4.15–5.02	1.39	2.32	0.91–1.88
	Practising evidence-based medicine and clinical guidelines	6.68	0.89	6.50–6.87	6.14	1.18	5.90–6.39	0.54	1.52	0.22–0.85
	Writing medico-legal reports or legal medicine or medical ethics	6.18	1.34	5.90–6.45	4.35	1.88	3.96–4.74	1.82	2.12	1.38–2.27
	Writing and publishing your research	6.00	1.64	5.66–6.34	2.93	1.98	2.52–3.35	3.07	2.52	2.54–3.59
	Designing, supervising, and managing research projects	5.76	1.79	5.38–6.13	2.92	2.11	2.48–3.36	2.84	2.54	2.31–3.36
	Accessing research resources, for example, time, money (funding), information	5.89	1.72	5.53–6.25	2.81	2.05	2.39–3.24	3.08	2.70	2.51–3.64
	Total	6.19	1.09	5.97–6.42	4.26	1.24	3.99–4.52	1.94	1.69	1.58–2.29

CI, confidence interval; s.d., standard deviation; CPD, continuing professional development; UMPDC, Uganda Medical and Dental Practitioners’ Council.

The domain with the highest training need for the doctors was research and audit, with a mean score (s.d.) of 1.94 (±1.69), followed by administration at 1.58 (±1.61) and clinical tasks at 1.28 (±1.29).

Assessment of the individual skilled activities revealed the top three CPD training needs in the research and audit domain: accessing research resources, writing and publishing research findings, and designing, supervising and managing research projects. Using new media and digital technology and good record keeping or data input also scored high on the CPD training needs under the administration domain. In the clinical tasks domain, the CPD training need scores were highest in evaluating patients’ psychological and social needs and doing procedures using new equipment and technology. Resource management or business skills or generating income or practice management and stress management scored highest for CPD training needs in the management and supervisory domain. The highest CPD training needs score in the communication and teamwork domain was for negotiation and conflict resolution skills.

Table 2 summarises the suggested specific training needs for CPD from the qualitative section of the questionnaire. There were 454 topics listed and grouped into: the clinical tasks 193/454 (42.5%); research and audit 97/454 (21.4%); management or supervisory tasks 82/454 (18.1%); communication and teamwork 41/454 (9.0%); and administration 41/454 (9.0%) domain. The clinical tasks domain had the most suggested CPD topics, while the communication and administration domains had the least number of suggested CPD topics.

**TABLE 2:** Participants suggested topics for continuing professional development.

Category	Suggested topics for CPD (*N* = 91)	Frequency	%
Clinical tasks	Use of modern and digital equipment or technology in health, minimal access surgery, laparoscopy, ultrasound	49	53.8
	Emergency recognition and management, basic life support (BLS), advanced trauma life support (ATLS), advanced life support (ALS), comprehensive emergency obstetric and neonatal care (CEMONC)	30	32.9
	Procedural skills and general surgical techniques	30	32.9
	Guidelines on managing common conditions (HIV, hypertension, diabetes, skin conditions, hepatitis, childhood illnesses)	27	29.7
	Evaluating patients’ psychological and social needs	9	9.9
	Preventive and health promotion medicine (role of physiotherapy and nutrition, infection control)	8	8.8
	Obstetric and gynaecologic management in COVID-19 and sickle cell disease	7	7.7
	Safety measures and management of COVID-19	7	7.7
	Interpretation of investigation results from ECG, X-rays, laboratory	5	5.5
	Good prescribing habits or evidence-based prescriptions	4	4.4
	Newborn care	4	4.4
	Mental health	4	4.4
	Oncology and palliative care	4	4.4
	Managing infertility	2	2.2
	Orientation in biomedical technology	2	2.2
	Nutritional rehabilitation	1	1.1
Research and audit	Research writing, designing, conducting and publishing	48	52.7
	Medico-legal report writing and issues (postmortem)	22	24.2
	Accessing research resources (information, time, grants)	18	19.8
	Designing quality improvement projects	9	9.9
Management or supervisory tasks	Leadership skills, management and governance	30	32.9
	Human and resource management in relation to ministry of planning standing orders	23	25.3
	Managing stress	11	12.1
	Government financial management systems	5	5.5
	Record keeping	5	5.5
	Data management	3	3.3
	Electronic patient information storage system, for example, health management information system (HMIS)	3	3.3
	Customer care management skills	2	2.2
Communication and teamwork	Health IT skills or e-learning or accessing online health resources	15	16.5
	Patient advocacy and social duty as a doctor and motivation	8	8.8
	Communication with colleagues, patients, and community, referral procedures	8	8.8
	Negotiation and conflict resolution and emotional intelligence	8	8.8
	Giving tutorials to students and conducting continuing medical education sessions	2	2.2
Administration	Business or entrepreneurship skills	20	21.9
	Public and hospital administration, for example, introducing new ideas or change at work	11	12.1
	Preparing executive reports or report writing	4	4.4
	Time management	3	3.3
	Budgeting and healthcare financing	2	2.2
	Handling political influences	1	1.1

COVID-19, coronavirus disease 2019; CPD, continuing professional development.

## Discussion

This study provides insight into the CPD training needs of doctors working in primary care settings in central Uganda. They primarily included: (1) research skills, (2) use of new media and digital technology, (3) clinical skills, (4) resource and stress management skills, (5) negotiation and (6) conflict resolution skills.

Research is a neglected but important skill in primary care that needs to be supported in this setting. Although most undergraduate and postgraduate curricula for doctors introduce research, primary care work settings have limited or no efforts in place to further sustain and nurture these skills. The study revealed that primary care doctors need to be continuously supported in their workspaces to identify operational researchable problems for which they can contribute local and practical solutions. The research skills would also empower the doctors to develop proposals worth competing for the available funding opportunities and improve work practices in their facilities. This finding is in agreement with an earlier conducted situation analysis in Uganda and Malawi.^27^ Improving work practices and facilities would in the long run result into job satisfaction and retention as well.

Some of the CPD training needs may have been largely influenced by the coronavirus disease 2019 (COVID-19) pandemic prevailing at the time of the study, for instance, the use of new media and digital technology. The health sector among others, was forced to learn and reinforce effective alternative ways of delivering services.^28^ This created challenges for the primary care doctors because they are the primary care team leaders.^11,12^ Therefore, the doctors had to think innovatively with no formal training to ensure that the primary care team is supported to continue service delivery.^29,30^ Consistent with the situated theory of learning,^31^ these challenges have created a CPD training need for the doctors to get better prepared. In a similar setting elsewhere, the primary care doctors sought innovative solutions with their patients on using digital technology to enhance service delivery.^32^

Notably, the findings from this study indicate that clinical skills are an important CPD training need for primary care doctors. The explanation may be because of the fact that clinical competence is a major expectation of the primary care doctor, among other responsibilities.^12,22^ Interestingly, the clinical skill of evaluating patients’ psychological and social needs scored highest for CPD training. The high CPD training need may be a result of the curricula for doctors in this setting that promotes the biomedical and not the biopsychosocial model of approach to patient care.^33^ The lack of this skill may also explain the frequent malpractice reports to the UMDPC because of violation of patients’ rights.^19^ Therefore, its emphasis during CPD may allow primary care doctors to effectively reflect on all their experiences and forge practical ways forward as teams. Further still, procedural skills were identified as an important area for CPD training. This may be explained by the broadness of primary care services and the constant development of new efficient methods of performing procedures. In return, there is a constant strain on the primary care doctors to ensure that they better themselves for the market or patients’ needs.

Management is a key domain in quality primary care services^34^ that is given no or limited attention in training programmes that produce primary care doctors.^35^ In this study, there were CPD training needs that indicated gaps in the management of self and the different resources at the workplace. These were stress and practice management, respectively. Failure to manage oneself results in stress or burnout, for which training in stress management has been recommended, among other strategies.^36^ The current deployment of at least two doctors at the HC IVs, in this study setting, was to partially address the risk of burnout. Effective management of stress would also contribute to job satisfaction and retention.^37^

This study, among others,^38,39^ has emphasised the importance of communication and teamwork skills for primary care doctors. These skills contribute to the formation of relationships, which are key in the communities of practice where the primary care doctors grow professionally.^31^ The CPD training needed for negotiation and conflict resolution skills was important to observe in this skill set. These skills are important for doctors in large, complex organisations such as the district healthcare system. In such a healthcare system, the doctors are expected to lead and function in a multidisciplinary team^12^ therefore, the need for continued development of such skills.

The strengths in this study included: use of a data collection questionnaire that has already been widely used in similar settings, homogeneity of study participants who were all medical doctors with at least a first degree in medicine (MBChB or its equivalent), registered with the UMDPC, and employed under the local government policy.

### Limitations of the study

The study used a self-reported questionnaire, the Hennessy-Hicks TNA, that may have affected data accuracy. A variation in the mode of delivery of the questionnaires, could have affected the quality of the collected responses. Nevertheless, all participants were given weekly reminders to complete the questionnaire to improve the response rate. Consecutive sampling is a non-probability method that could have introduced selection bias. The study was carried out only in the central region and therefore, the findings may not be generalisable to other regions in the country. Data collection was performed during the COVID-19 pandemic when doctors had more workload affecting their availability for the study. This could have affected and influenced the quality of the data collected.

### Recommendations

The accredited CPD providers in this region could focus on the CPD training needs identified in this study to remain relevant and responsive to primary care physicians’ needs. Further research is needed to identify ongoing activities in the district health system where CPD training gaps identified in this study could be addressed. It is also important to further investigate strategies to continue applying research skills acquired during the formal training of doctors. This must be coupled with continued advocacy for primary healthcare funds to support the formation and implementation of a primary care research agenda at the district level. These efforts may contribute to a holistic improvement of primary care services in the district health system and the individual primary care doctors. Keeping up to date with new media and digital technology is recommended for CPD because of the continuously changing times that may affect the delivery of primary care services. Training in stress and practice management could be incorporated into the CPD sessions for primary care doctors to improve performance. In addition, other non-medical skills such as negotiation and conflict resolution need attention through CPD.

## Conclusion

In order to effectively support the ongoing CPD efforts, this study has identified CPD training needs for primary care doctors that include: research skills, use of new media and digital technology, clinical skills, resource and stress management skills, and negotiation and conflict resolution skills.

The CPD training needs identified in each of the five domains provide evidence for better planning by the CPD providers and resource allocation by the facility and district health managers. A training needs analysis for primary care doctors would benefit from periodic updates because of the evolving professional demands and pattern of healthcare needs in the work environment.
